# Chitinases as a potential diagnostic and prognostic biomarker for amyotrophic lateral sclerosis: a systematic review and meta-analysis

**DOI:** 10.1007/s10072-024-07301-5

**Published:** 2024-01-09

**Authors:** Aoling Xu, Yujun Luo, Yudi Tang, Fen Yang, Xiaolian Gao, Guiyuan Qiao, Xinhong Zhu, Jing Zhou

**Affiliations:** 1 School of Acupuncture-Moxibustion and Orthopedics, Hubei University of Chinese Medicine, Wuhan, 430065 China; 2 Department of Tuina and Rehabilitation Medicine, Hubei Provincial Hospital of Traditional Chinese Medicine, Wuhan, 430061 China; 3 Department of Tuina and Rehabilitation Medicine, Affiliated Hospital of Hubei University of Chinese Medicine, Wuhan, 430061 China; 4 Department of Tuina and Rehabilitation Medicine, Hubei Provincial Institute of Traditional Chinese Medicine, Wuhan, 430061 China; 5 First Clinical Medical College, Hubei University of Chinese Medicine, Wuhan, 430065 China; 6 School of Nursing, Hubei University of Chinese Medicine, Wuhan, China

**Keywords:** Amyotrophic lateral sclerosis, CHIT1, CHI3L1, CHI3L2, Biomarker, Cerebrospinal fluid, Blood

## Abstract

Amyotrophic lateral sclerosis (ALS) is a fatal neurodegenerative disease characterized by the degeneration of motor neurons, and there is currently a lack of reliable diagnostic biomarkers. This meta-analysis aimed to evaluate CHIT1, CHI3L1, and CHI3L2 levels in the cerebrospinal fluid (CSF) or blood and their diagnostic potential in ALS patients. A systematic, comprehensive search was performed of peer-reviewed English-language articles published before April 1, 2023, in PubMed, Scopus, Embase, Cochrane Library, and Web of Science. After a thorough screening, 13 primary articles were included, and their chitinases-related data were extracted for systematic review and meta-analysis. In ALS patients, the CSF CHIT1 levels were significantly elevated compared to controls with healthy control (HC) (SMD, 1.92; 95% CI, 0.78 – 3.06; *P* < 0.001). CHIT1 levels were elevated in the CSF of ALS patients compared to other neurodegenerative diseases (ONDS) control (SMD, 0.74; 95% CI, 0.22 – 1.27; *P* < 0.001) and exhibited an even more substantial increase when compared to ALS-mimicking diseases (AMDS) (SMD, 1.15; 95% CI, 0.35 – 1.94, *P* < 0.001). Similarly, the CSF CHI3L1 levels were significantly higher in ALS patients compared to HC (SMD, 3.16; 95% CI, 1.26 – 5.06, *P* < 0.001). CHI3L1 levels were elevated in the CSF of ALS patients compared to ONDS (SMD, 0.75; 95% CI, 0.32 – 1.19; *P* = 0.017) and exhibited a more pronounced increase when compared to AMDS (SMD, 1.92; 95% CI, 0.41 – 3.42; *P* < 0.001). The levels of CSF chitinases in the ALS patients showed a significant increase, supporting the role of CSF chitinases as diagnostic biomarkers for ALS.

**Supplementary Information:**

The online version contains supplementary material available at 10.1007/s10072-024-07301-5.

## Introduction

Amyotrophic lateral sclerosis (ALS) is a heterogeneous neurodegenerative disease that is characterized by the degeneration of both upper and lower motor neurons [1]. It begins insidiously with focal weakness but spreads relentlessly to involve most muscles, including the diaphragm. Typically, mortality resulting from respiratory paralysis transpires within a span of 3 to 5 years [2]. ALS is a relatively uncommon disease, with a standardized global incidence rate of merely 1.68 per 100,000 person-years of follow-up, as determined through meta-analysis [3]. Furthermore, ALS incidence varies by sex, with an overall standardized male-to-female ratio of 1.35, which is influenced by age at onset [4]. While it is relatively uncommon, its impact on affected individuals and their families is profound. Further research is crucial to identify more effective treatments and interventions to prolong survival and enhance the quality of life for ALS patients.

The clinical, genetic, and neuropathological heterogeneity, along with similarities to other neuromuscular disorders, especially in the early stages of the disease, are often described as mimicking symptoms of ALS, which frequently necessitate the application of additional diagnostic methods in clinical practice [5]. Despite considerable efforts to enhance the sensitivity of diagnostic criteria, the delay from symptom onset to diagnosis remains between 8 and 15 months [6], which is deemed unacceptable given the brief survival time associated with the disease. However, biomarkers in both CSF and peripheral blood can play a role in the early diagnosis and treatment of ALS, as these biomarkers may appear during the onset, progression, and prognosis stages of the disease [7]. As a consequence, a multitude of biomarker-based studies have emerged, including neurofilaments [8–11], chitinases [12, 13], TDP-43 [14], urinary neopterin [15], cystatin c [7], creatine kinase [16], tau [17], and other biomarkers [18] with potential diagnostic value, to assist in clinical diagnosis and aid in estimating prognosis in ALS. Although significant efforts have been made in biomarker research and several candidate molecules have been identified, which have repeatedly demonstrated their ability to reflect disease invasiveness or prognosis, they have not yet reached routine clinical application [19]. Therefore, further research on promising biomarkers is urgently needed to serve as tools for reducing diagnostic delay in ALS, evaluating prognosis, and conducting clinical therapeutic and preventive trials.

Chitinases are enzymes known as glycosyl hydrolases. Although mammals cannot synthesize or utilize chitin as a nutrient, the human genome encodes eight members of the GH18 family, including chitinases, chitotriosidase (CHIT1), and acid mammalian chitinase (AM Case), as well as several CLPs, such as chitinase 3-like 1 (CHI3L1) and chitinase 3-like 2 (CHI3L2) [20]. Prior research has suggested chitinases’ involvement in the development of diverse human fibrotic and inflammatory conditions, notably respiratory ailments, gastrointestinal issues, and neurological disorders [21–23]. Despite a limited understanding of chitinases’ physiological and pathophysiological functions, they are increasingly acknowledged as biomarkers across various neurological disorders. Frequently, chitinases levels measured in CSF correlate with disease activity and progression [24]. Findings demonstrated that CHIT1 and CHI3L2 are associated with the rate of disease progression in ALS and serve as independent prognostic factors for survival [25–28].

According to the current research evidence, chitinases may be a potential biomarker for ALS. However, there is still a lack of sufficient investigation to determine the use of chitinases levels for the diagnosis or prognosis of ALS. The objective of this research is to systematically examine all investigations that analyze the concentrations of chitinases in the CSF and blood of individuals diagnosed with ALS. Additionally, a meta-analysis will be conducted to explore whether a significant distinction exists in the concentrations of CSF and blood chitinases between ALS patients and HC, ONDS, and AMDS control subjects.

## Methods

The investigation adhered to the guidelines of the Preferred Reporting Items for Systematic Reviews and Meta-Analyses (PRISMA) during its course [29] and has been registered in PROSPERO(CRD42023412867).

### Search strategy and study selection

A systematic search was conducted for peer-reviewed English articles published until April 1, 2023, on PubMed, Scopus, Embase, Cochrane Library, and Web of Science. The study investigated CSF and blood chitinases as a biomarker for ALS. Details of the search strategy are provided in Table 1. Two reviewers (ALX and XHZ) conducted an assessment of the literature screening process to verify compliance with the inclusion/exclusion criteria. The ultimate determination of article inclusion within our study was reached through consensus among ALX, XHZ, YJL, and JZ.

**Table 1:** Search strategy of PubMed

Search number	Query	Results
#1	“Amyotrophic Lateral Sclerosis”[Mesh]	23472
#2	((((“Amyotrophic Lateral Sclerosis”[Mesh]) OR (Lou Gehrig’s Disease[Title/Abstract])) OR (Amyotrophic Lateral Sclerosis[Title/Abstract])) OR (Motor Neuron Disease[Title/Abstract])) OR (ALS[Title/Abstract])	43673
#3	#1 OR #2	43673
#4	“Chitinases”[Mesh]	5191
#5	((((“Chitinases”[Mesh]) OR (Chitinases [Title/Abstract])) OR (Chitinase[Title/Abstract])) OR (Endochitinase[Title/Abstract]))	8897
#6	“chitotriosidase” [Supplementary Concept]	469
#7	“Chitinase-3-Like Protein 1”[Mesh]	1320
#8	((((((“Chitinase-3-Like Protein 1”[Mesh]) OR (Chitinase 3 Like Protein 1[Title/Abstract])) OR (Cartilage Glycoprotein 39[Title/Abstract])) OR (GP-39 Protein[Title/Abstract])) OR (YLK-40 Protein[Title/Abstract])) OR (CHI3L1 Protein[Title/Abstract])) OR (CGP-39 Protein[Title/Abstract])	1521
#9	“CHI3L2 protein, human” [Supplementary Concept]	33
#10	(((“CHI3L2 protein, human” [Supplementary Concept]) OR (CHI3L2 protein, human[Title/Abstract])) OR (chitinase 3-like 2 protein, human[Title/Abstract])) OR (YKL-39 protein, human[Title/Abstract])	33
#11	#4 OR #5 OR #6 OR #7 OR #8 OR #9 OR #10	9243
#12	#3 AND #11	45

Inclusion criteria: (1) The study assessed the association between levels of chitinases in the CSF or blood of ALS patients and controls; (2) the study compared CSF or blood levels of any of CHIT1, CHI3L1, and CHI3L2 in ALS patients and control patients and reported mean or median, quartiles of CSF or blood CHIT1, CHI3L1, and CHI3L2 levels or upper and lower bounds; (3) provided a demographic description of the patients. Exclusion criteria: (1) Only studies performed on animal experiment; (2) studies that evaluated chitinases in samples other than CSF and blood, such as the spinal cord; (3) the study employed non-quantitative methods to estimate the concentration of chitinases; (4) reviews, conference, and meta-analysis.

### Data extraction and methodological quality

Two authors independently extracted the following elements from the incorporated studies: author name, publication year, country of origin, the sample size for ALS cases and control groups, mean and standard deviation of levels of CSF CHIT1, CHI3L1, and CHI3L2, mean age, mean male ratio, disease duration, analysis technique, the disease severity (ALSFRS-R), AUC (area under curve). Since several studies reported median and interquartile range (median (IQR)) levels of chitinases, these were converted to mean and SD using the method proposed by Hozo et al. [30], Luo et al. [31], and Wan et al. [32].

The Newcastle–Ottawa Scale (NOS) criteria were utilized to evaluate the quality and risk of bias in all the articles encompassed in this study. Nine studies were found to exhibit a low bias risk, while four studies were identified with a high bias risk. (Table 2). The evaluation was conducted independently by two authors (JZ and YJL). Any disagreements were resolved through consensus among ALX, XHZ, YJL, and JZ.

**Table 2:** Newcastle–Ottawa quality assessment scale of case and control studies

Study		Selection			Comparability		Exposure		Total
	1	2	3	4		1	2	3	
Abu-Rumeileh, 2020	☆	☆	–	☆	☆	☆	☆	☆	7
Andres-Benito, 2018	☆	☆	–	☆	☆	☆	☆	☆	7
Costa, 2021	☆	–	–	☆	☆	☆	☆	☆	6
Gille, 2019	☆	☆	–	☆	☆	☆	☆	☆	7
Haji, 2022	☆	–	–	☆	☆	☆	☆	☆	6
Illán-Gala, 2018	☆	☆	–	☆	☆	☆	☆	☆	7
Masrori, 2022	☆	☆	–	☆	☆	☆	☆	☆	7
Steinacker, 2021	☆	–	–	☆	–	☆	☆	☆	5
Steinacker, 2018	☆	☆	–	☆	☆	☆	☆	☆	7
Thompson, A, 2019	☆	☆	–	☆	☆	☆	☆	☆	7
Varghese, A, 2020 Varghese, A, 2013	☆ ☆	☆ –	– –	☆ ☆	☆ ☆	☆ ☆	☆ ☆	☆ ☆	7 6
Verde, F, 2021	☆	☆	–	☆	☆	☆	☆	☆	7

☆ The item is credited with one point out of nine

### Assessment of evidence quality

Two separate investigators utilized the Grading of Recommendations Assessment, Development and Evaluation (GRADE) [33] methodology to appraise the comprehensive evidence quality for each outcome. This process aimed to gauge the certainty of the evidence. All these procedures were carried out through the employment of the GRADEpro software.

### Statistical analysis

All statistical analyses were performed using STATA 16 software. The outcome measures were measured in standardized mean differences (SMD). Using a random effects model, the Cohen’s d-statistic was utilized to compare the SMD, considering the bias from tiny sample sizes. SMDs were reported as odds with 95% confidence intervals. The heterogeneity in all outcome measures was gauged employing *I*^2^ -values. Publication bias detection was performed by visually inspecting funnel plot asymmetry and employing Begg’s test. Sensitivity analysis was utilized to assess the sources of heterogeneity. We conducted sub-group research based on predetermined factors: age, diagnostic criteria, control group age matching, and male rates. A random effects model meta-regression analysis was employed to analyze these factors comprehensively. The statistical significance of this meta-analysis was set at *P* value < 0.05 unless stated otherwise.

## Results

### Summary of included research

**Fig. 1:**
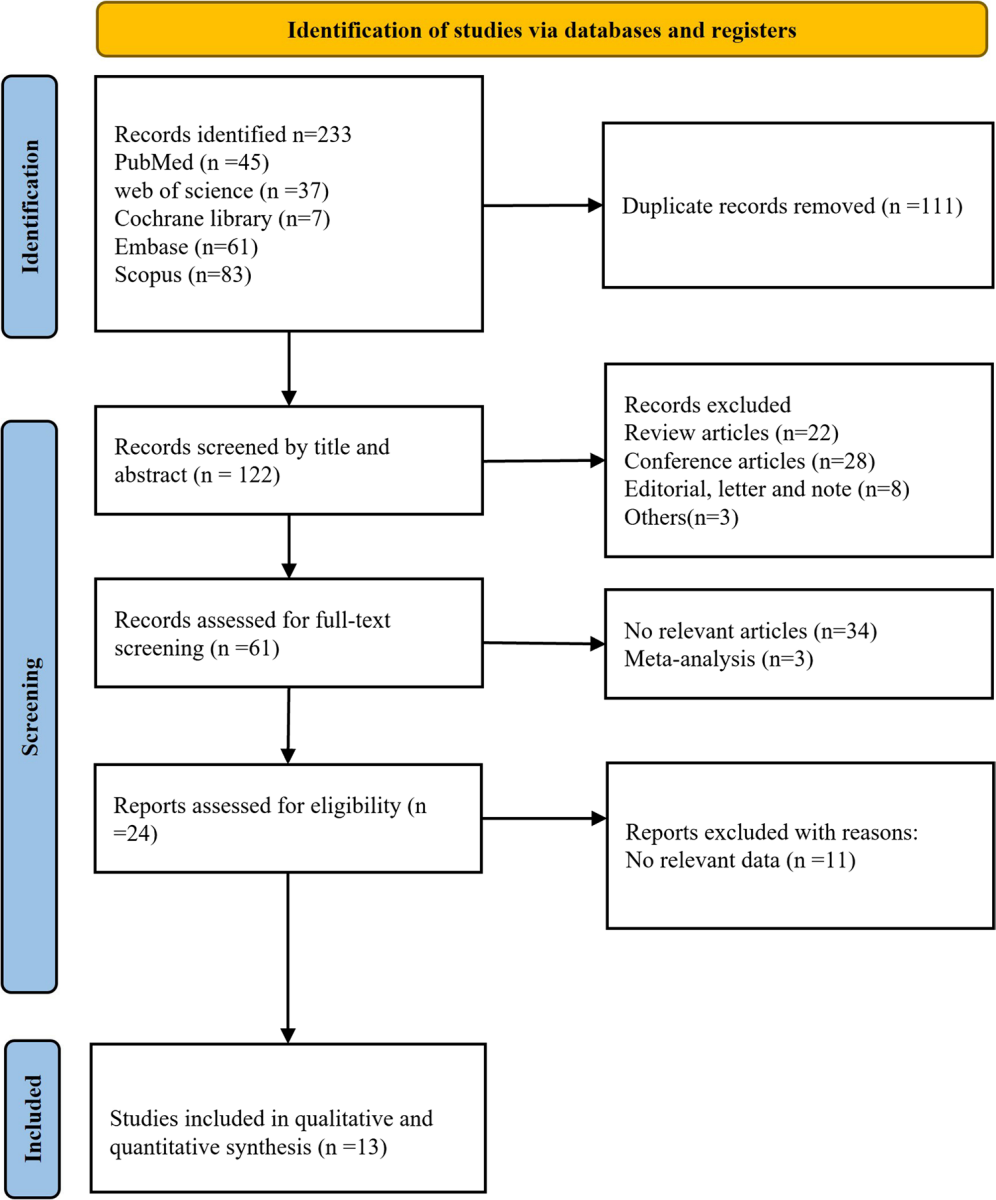
PRISMA flowchart describing the study selection

The literature search yielded a total of 233 papers from electronic databases. Among these articles, 111 duplicates were excluded. After assessing the titles and abstracts, 61 citations were removed for various reasons, leaving 61 papers for comprehensive evaluation through full-text review. Finally, 13 original studies were identified in the meta-analysis [12, 25–41]. The detailed process can be seen in Fig. 1. All studies aimed to investigate CSF or blood chitinases levels between ALS patients and controls. Twelve of these studies measured chitinases concentrations in CSF or blood by ELISA and one by ECL immunoassay. Among them, ten studies extracted CSF CHIT1, seven studies extracted CSF CHI3L1, two studies extracted CSF CHI3L2, and two studies extracted serum CHI3L1. The comprehensive characteristics of the studies were summarized in Table 3.

**Table 3:** The detailed characteristics of the included studies

Study	Country	Groups	N (ALS/OND/NND)	Gender (% male) (ALS/OND/NND)	Age (ALS/OND/NND)		Disease duration (months)	Diagnosis	Analysis technique	ALSFRS-R	AUC
Abu-Rumeileh, 2020	Italy	ALS/HC/OND	80/43/46	57.5/51.2/67.4	62.21 ± 12.41/63.93 ± 10.60/62.37 ± 11.93	28 ± 28		El Escorial	ELISA	39 ± 6	ALS/OND: 0.796 (CHIT1), 0.74(CHI3L1); ALS/HC: 0.817 (CHIT1), 0.879 (CHI3L1)
Andres-Benito, 2018	Spain	ALS/HC	86/21	54.7/42.9	62.5/NA	NA		Clinical diagnosis	ELISA	NA	ALS/HC: 0.6254 (CHI3L1)
Costa, 2021	Portugal	ALS/OND	34/24	73.5/50	56.81 ± 14.63/54.75 ± 17.65	NA		El Escorial	ELISA	NA	ALS/OND: 0.8 (CHIT1)
Gille, 2019	Belgium	ALS/OND	105/102	57/48	62.67 ± 9.54/54.61 ± 13.77	13.39 ± 12.32		El Escorial and Awaji	ELISA	37.35 ± 6.36	ALS/OND: 0.78 (CHIT1), 0.76 (CHI3L1)
Haji, 2022	Japan	ALS/HC/OND	32/41/8	62.5/41.4/87.5	69.36 ± 8.54/63.10 ± 13.06 /65.99 ± 6.9	8.36 ± 5.43		proposed criteria	ECL immunoassay	43.8 ± 3.49	NA
Illán-Gala, 2018	Spain	ALS/HC/OND	38/49/86	47.3/53.1/62.8	66.6/64.2/NA	NA		El Escorial	ELISA	NA	NA
Masrori, 2022	Belgium	ALS/OND	83/49	58/47	62.68 ± 9.25/49.71 ± 13.42	12.59 ± 8.43		El Escorial and Awaji	ELISA	38.05 ± 6.37	ALS/OND: 0.89 (CHIT1), 0.88 (CHI3L1)
Steinacker, 2021	Europe	ALS/HC/OND/AMD	161/43/30/42	64/35/73/71	61.8 ± 9.58/52.0581 ± 10.756/58.2163 ± 14.4588/55.32 ± 12.18	12.67 ± 14.46		Proposed criteria	ELISA	41.7 ± 7.89	ALS/HC: 0.86 (CHIT1); ALS/OND: 0.76 (CHIT1)
Steinacker, 2018	Germany	ALS/AMD/OND	60/46/25	65/76/32	61.44 ± 13.67 /58.71 ± 19.9/68.9266 ± 8.6473	53.24 ± 22.7		NA	ELISA	40.83 ± 3.78	ALS/HC: 0.8567 (CHIT1)
Thompson, A, 2019	UK	ALS/HC/AMD	82/25/12	76.8/48/91.7	56.8 ± 11.2 /56.5 ± 9.2/57.7 ± 15.5	NA		Neurologist diagnosis	ELISA	NA	ALS/OND: 0.84 (CHIT1), 0.73 (CHI3L1), 0.88 (CHI3L2); ALS/HC: 0.92 (CHIT1), 0.80 (CHI3L1), 0.9 (CHI3L2)
Varghese, A, 2020	Indian	ALS/HC	158/48	77.2/62.5	50.82 ± 9.57/42.88 ± 8.25	14.55 ± 12.96		El Escorial	ELISA	28.37 ± 7.28	ALS/HC: 0.877 (CHIT1)
Varghese, A, 2013	Indian	ALS/HC	16/10	62.5/84.6	47.38 ± 5.38/45.7 ± 7.04	14.19 ± 10.59		NA	ELISA	NA	NA
Verde, F, 2021	Italy	ALS/AMD	28/10	57.1/50	59.0359 ± 10.7019/59.1698 ± 14.6201	11.3924 ± 8.2022		El Escorial	ELISA	40.1785 ± 4.2964	ALS/AMD: 0.740 (CHIT1)

*HC* health control, *OND* other neurodegenerative diseases, *AMD* ALS-mimicking diseases, *AUC* area under curve (important indicators of receiver operating characteristic curves)

### CHIT1 levels in CSF

**Fig. 2:**
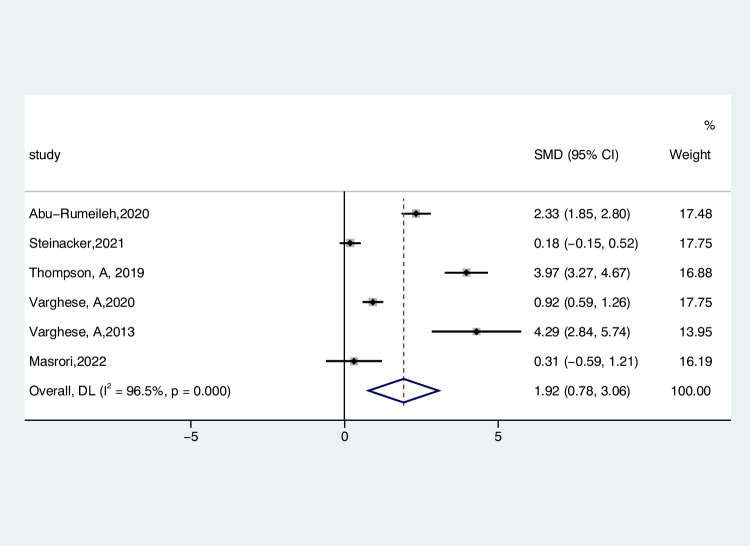
Forest plot of CHIT1 levels in CSF of ALS patients versus HC

The findings extracted from 6 studies underwent analysis using a random-effects framework to contrast the CSF CHIT1 levels between individuals with ALS and those with HC. The dataset included 580 ALS patients and 174 control subjects. As shown in Fig. 2, the CSF CHIT1 level in patients with ALS was significantly higher than that in the HC (ALS-C pooled SMD, 1.92; 95% CI, 0.78 – 3.06; *P* < 0.001), and a notable degree of heterogeneity was observed (*I*^2^ = 96.5%, *P* < 0.001). The examination of the funnel plot for ALS-C revealed an absence of noteworthy publication bias, a finding that was substantiated by the results of the Begg’s test (*P* = 0.707). In the sensitivity analysis of these studies. The study by Thompson et al. in 2019 tended to have considerable heterogeneity, which significantly impacted the aggregate effect size estimates (Figure S1). After excluding this study, the heterogeneity decreased slightly (*I*^2^ = 94.7%, *P* < 0.001). In the EI diagnostic criteria subgroup, the heterogeneity effect decreased to 92.8% (Figure S2), with no significant change in the remaining subgroups. To further investigate the impact of heterogeneity, we conducted a meta-regression analysis of these three factors. The results showed that age (*P* = 0.429), diagnostic criteria (*P* = 0.732), and age-matched status of controls (*P* = 0.720) were not sources of heterogeneity (Figure S3).

**Fig. 3:**
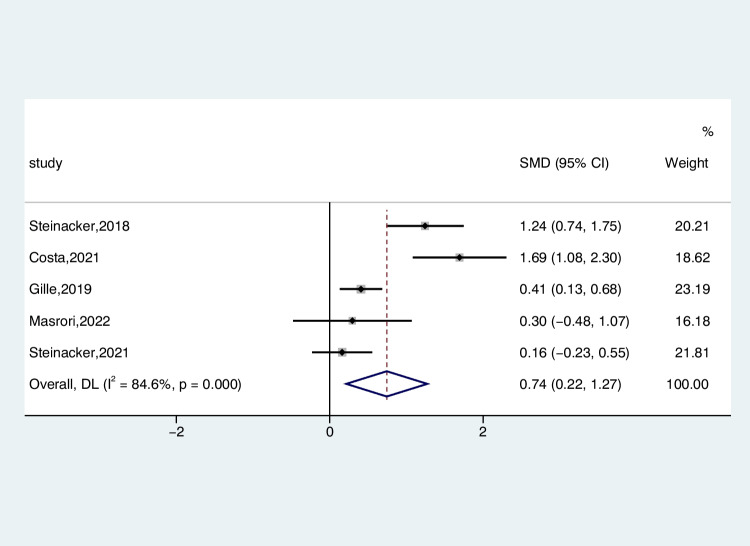
Forest plot of CHIT1 levels in CSF of ALS patients versus ONDS

The dataset, comprising 443 ALS patients and 188 control subjects, was analyzed to compare CSF CHIT1 levels between individuals with ALS and those with ONDS across findings extracted from 5 studies. CHIT1 levels were elevated in the CSF of ALS patients compared to ONDS patients. (ALS-C pooled SMD, 0.74; 95% CI, 0.22 – 1.27; *P* < 0.001). The heterogeneity of ONDS decreased as a control compared to normal subjects as a control (*I*^2^ = 84.6%, *P* < 0.001) (Fig. 3). The examination of the funnel plot of ALS-C revealed no significant publication bias, as confirmed by the results of Begg’s test (*P* = 0.462). In the sensitivity analysis of these studies, the Costa et al. studies tended to have considerable heterogeneity, significantly impacting the aggregate effect size estimates (Figure S4). After excluding this study, heterogeneity decreased to 74.7% (*P* = 0.008) (Figure S5).

**Fig. 4:**
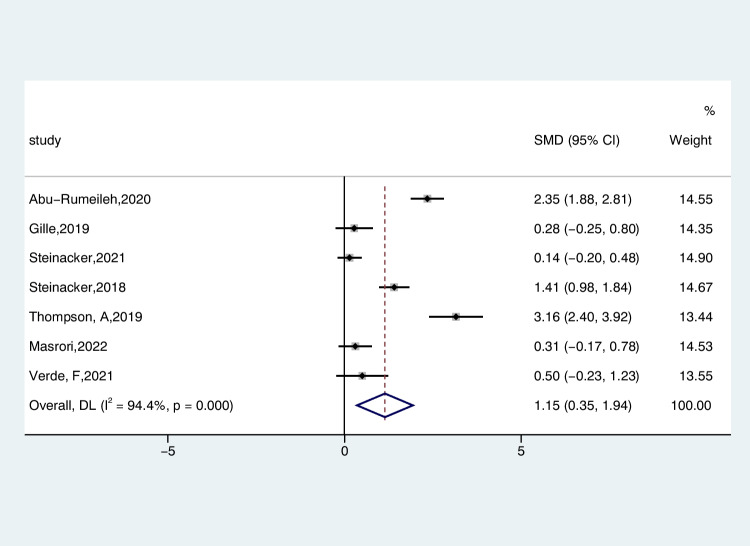
Forest plot of CHIT1 levels in CSF of ALS patients versus AMD

Seven studies reported CSF CHIT1 levels in 599 patients with ALS and 194 patients with AMD, comparing the differences by random-effects modeling. The CSF CHIT1 level in ALS patients exhibited a notable increase compared to that observed in individuals with AMD (ALS-C pooled SMD, 1.15; 95% CI, 0.35 – 1.94; *P* < 0.001). A notable degree of heterogeneity was observed (*I*^2^ = 94.4%, *P* < 0.001) (Fig. 4). The examination of the funnel plot for ALS-C revealed an absence of noteworthy publication bias, a finding that was substantiated by the results of the Begg’s test (*P* = 0.368). In the sensitivity analysis of these studies, the Thompson et al. studies tended to have considerable heterogeneity, significantly impacting the aggregate effect size estimates (Figure S6). After we excluded their studies, the heterogeneity decreased slightly (*I*^2^ = 93.2%, *P* < 0.001). Heterogeneity did not change significantly after subgroup analysis with age, diagnostic criteria, and male rate as factors in the ALS group. Similarly, a meta-regression analysis was performed on these three factors. The results showed that age (*P* = 0.464), diagnostic criteria (*P* = 0.572), and gender (*P* = 0.510) were not sources of heterogeneity.

### CHI3L1 levels in CSF and serum

**Fig. 5:**
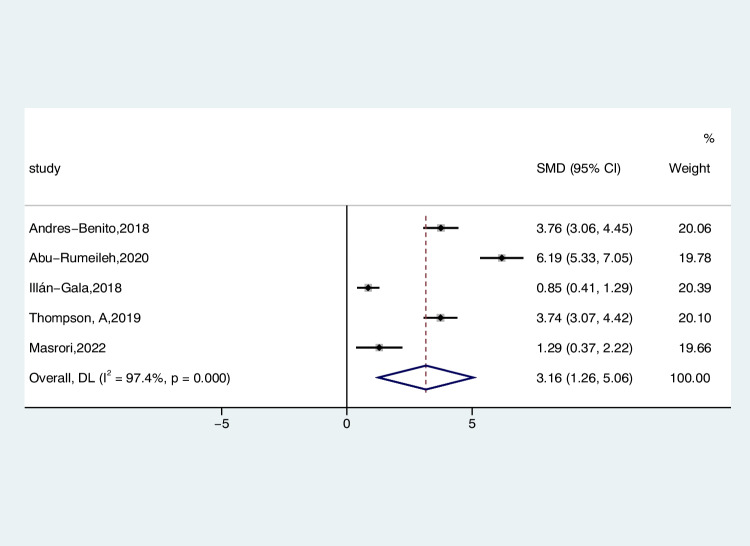
Forest plot of CHI3L1 levels in CSF of ALS patients versus HC

Five studies reported CSF CHI3L1 levels in 369 ALS patients and 143 healthy individuals, compared to the differences by random-effects modeling. The CSF CHI3L1 level in ALS patients exhibited a notable increase compared to the HC (ALS-C pooled SMD, 3.16; 95% CI, 1.26 – 5.06; *P* < 0.001) (Fig. 5) in the sensitivity analysis of these studies. The Illán-Gala et al. studies tended to have considerable heterogeneity, significantly impacting the aggregate effect size estimates (Figure S7). After we excluded them from the survey, heterogeneity decreased to 94.8% (*P* < 0.001) (Figure S8). Heterogeneity remained stable following subgroup analysis, considering diagnostic criteria and age-matched status of controls as covariates within the ALS group. Additionally, the meta-regression analysis revealed that neither diagnostic criteria (*P* = 0.664) nor age-matched status (*P* = 0.573) constituted a significant origin of heterogeneity.

**Fig. 6:**
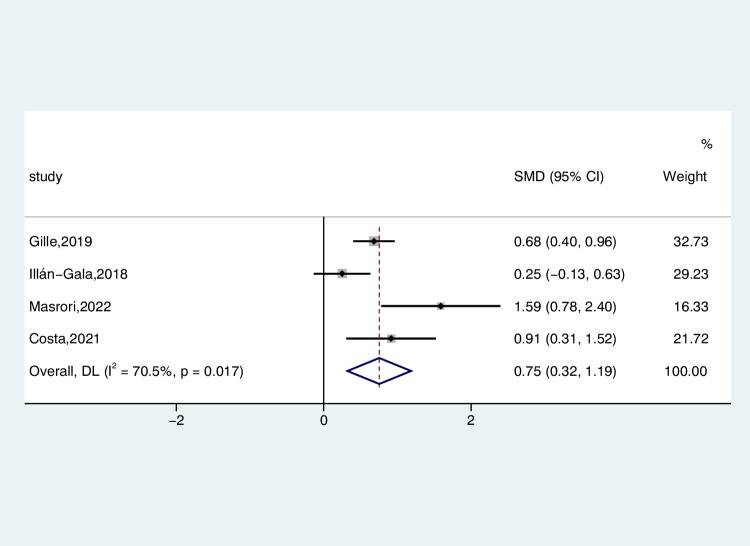
Forest plot of CHI3L1 levels in CSF of ALS patients versus ONDS

The results extracted from four studies were analyzed using a random-effects model to compare the levels of CSF CHI3L1 in 258 ALS patients and 213 ONDS patients (ALS-C pooled SMD, 0.75; 95% CI, 0.32 – 1.19; *P* = 0.017) (Fig. 6). Similarly, there is a significant reduction in the heterogeneity of ONDS compared to the normal control group (*I*^2^ = 70.5%, *P* = 0.017). The assessment of the funnel plot for ALS-C indicated the absence of significant publication bias, a conclusion supported by the outcomes of the Begg’s test (*P* = 0.308). We conducted a sensitivity analysis of these studies, demonstrating that the Illán-Gala et al. studies tended to have large heterogeneity, which significantly impacted the aggregate effect size estimates (Figure S9). After we excluded them from the study, heterogeneity decreased to 55.6% (*P* = 0.105) (Figure S10).

**Fig. 7:**
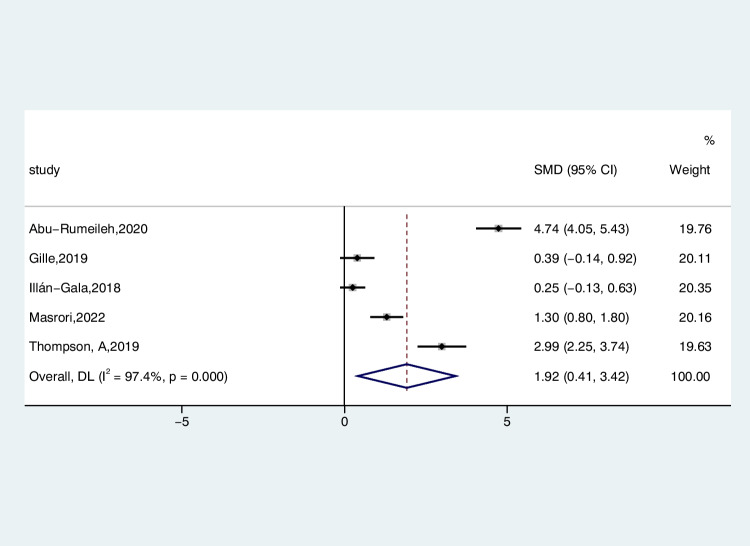
Forest plot of CHI3L1 levels in CSF of ALS patients versus AMDS

Five studies reported CSF CHI3L1 levels in 388 patients with ALS and 182 patients with AMDS, comparing the differences by random-effects modeling. The CSF CHI3L1 level in ALS patients exhibited a significant increase compared to the AMDS (ALS-C pooled SMD, 1.92; 95% CI, 0.41 – 3.42; *P* < 0.001) (Fig. 7). The examination of the funnel plot for ALS-C revealed no substantial evidence of publication bias, a conclusion corroborated by the results of the Begg’s test (*P* = 0.221). In the sensitivity analysis of the studies, considerable heterogeneity was observed in the study conducted by Abu-Rumeileh et al. in 2019, exerting a notable influence on the overall effect size estimates (Figure S11). After excluding this study, the heterogeneity decreased slightly (*I*^2^ = 93.7%, *P* < 0.001).

**Fig. 8:**
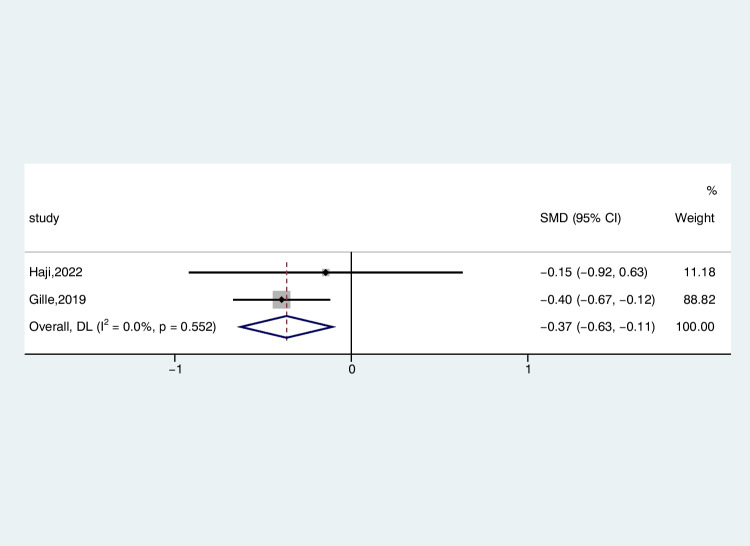
Forest plot of CHI3L1 levels in serum of ALS patients versus AMDS

Two studies reported serum CHI3L1 levels in 137 patients with ALS and 110 patients with AMDS, comparing the differences by random-effects modeling. Serum levels of CHI3L1 exhibited a reduction in ALS patients when contrasted with individuals with AMDS (ALS-C pooled SMD, − 0.37; 95% CI, − 0.63 to − 0.11; *P* = 0.552) (Fig. 8), but the difference was not statistically significant, possibly due to the limited inclusion of studies.

### GRADE analysis for the outcome

The scoring of confidence in outcome indicators used the GRADE grading scale. When using HC as the control, the evidence grade for CHIT1 and CHI3L1 levels in CSF is high. When ONDS is used as the control, the evidence grade for CHIT1 and CHI3L1 levels in CSF is low. When AMDS is used as the control, the evidence grade for CHIT1 levels in CSF is moderate, and for CHI3L1, it is high. In serum, the evidence grade for CHI3L1 is low when AMDS is the control. All results are presented in Table 4.

**Table 4:** GRADE analysis and certainty of evidence

Quality assessment							No. of patients		Effect		Quality	Importance
No. of studies	Design	Risk of bias	Inconsistency	Indirectness	Imprecision	Other considerations	Chitinase	Control	Relative (95% CI)	Absolute		
CHIT1 levels in CSF versus healthy control (measured with ELISA; better indicated by higher values)												
6	Observational studies	No serious risk of bias	Serious^1^	No serious indirectness	No serious imprecision	Very strong association^2^ reduced effect for RR > > 1 or RR < < 1^3^	580	174	-	SMD 1.92 higher (0.78 to 3.06 higher)	⊕⊕⊕⊕ High	Important
CHIT1 levels in CSF versus other neurodegenerative diseases (measured with ELISA; better indicated by higher values)												
5	Observational studies	No serious risk of bias	No serious inconsistency	No serious indirectness	No serious imprecision	None	443	188	-	SMD 0.74 higher (0.22 to 1.27 higher)	⊕⊕O Low	Important
CHIT1 levels in CSF versus ALS-mimicking diseases (measured with ELISA; better indicated by higher values)												
7	Observational studies	No serious risk of bias	Serious^1^	No serious indirectness	No serious imprecision	Strong association^4^ reduced effect for RR > > 1 or RR < < 1^3^	599	194	-	SMD 1.15 higher (0.35 to 1.94 higher)	⊕⊕⊕O Moderate	Important
CHI3L1 levels in CSF versus healthy control (measured with ELISA; better indicated by higher values)												
5	Observational studies	No serious risk of bias	Serious^1^	No serious indirectness	No serious imprecision	Very strong association^2^ reduced effect for RR > > 1 or RR < < 1^3^	369	143	-	SMD 3.16 higher (1.26 to 5.06 higher)	⊕⊕⊕⊕ High	Important
CHI3L1 levels in CSF versus other neurodegenerative diseases (measured with ELISA; better indicated by lower values)												
4	Observational studies	No serious risk of bias	No serious inconsistency	No serious indirectness	No serious imprecision	None	258	213	-	SMD 0.75 higher (0.32 to 1.19 higher)	⊕⊕OO Low	Important
CHI3L1 levels in CSF versus ALS-mimicking diseases (measured with ELISA; better indicated by lower values)												
5	Observational studies	No serious risk of bias	Serious^1^	No serious indirectness	No serious imprecision	Very strong association^2^ reduced effect for RR > > 1 or RR < < 1^3^	388	182	-	SMD 1.92 higher (0.41 to 3.42 higher)	⊕⊕⊕⊕ High	Important
CHI3L1 levels in serum versus ALS-mimicking diseases (better indicated by lower values)												
2	Observational studies	No serious risk of bias	No serious inconsistency	No serious indirectness	No serious imprecision	None	137	110	-	SMD − 0.37 lower (− 0.36 higher to − 0.11 lower)	⊕⊕OO Low	Important

^1^Higher heterogeneity between studies

^2^SMD ≥ 1.0 showed a very large difference between patients in the ALS group and those in the control group

^3^Amyotrophic lateral sclerosis is a disease of uncertain etiology, influenced by multiple factors, with a large heterogeneity of disease, influenced by multiple confounding factors

^4^SMD ≥ 1.0 showed a large difference between patients in the ALS group and the control group

## Discussion

Through comprehensive analysis, our study has revealed a significant elevation in chitinases enzyme levels in the CSF of ALS patients. This finding substantiates the role of CSF chitinases enzymes as diagnostic biomarkers for ALS.

Our meta-analysis indicates that measuring the concentration of CHIT1 in CSF can be utilized to differentiate between ALS and HC, ONDS, as well as AMDS. According to recent scientific research, there is a strong correlation between chitinases and neuroinflammation. Neuroinflammation plays a pivotal part in both the initial neuroprotective and subsequent neurotoxic stages of ALS pathogenesis [42]. CHIT1 is the first chitinases discovered and characterized in humans. It was initially detected in macrophages obtained from individuals with Gaucher disease [43, 44]. The enzyme is expressed in standard and pathological conditions, primarily by activated macrophages [45]. CHIT1 plays a vital role in the process of inflammation by serving as a defensive mechanism against chitin pathogens, thereby facilitating the innate immune response. The expression of CHIT1 is elevated in the microglia and macrophages present in the spinal cord of ALS patients and their CSF. This increased expression of CHIT1 is associated with the severity and progression of the disease [12]. In their study, Varghese et al. demonstrated that CHIT1 is an early diagnostic biomarker in sporadic ALS. They found that CHIT1 activates glial cells, which then acquire a toxic phenotype that results in neuroinflammation and ultimately leads to the death of motor neurons [38]. The results showed that for the CHIT1 content in CSF, the discriminatory ability between the ALS patient group and HC was better than that of the ONDS and AMDS control groups. Additionally, AMDS outperforms ONDS, demonstrating the potential of CHIT1 for the differential diagnosis of ALS and AMD, and indicating that CHIT1 may exert an impact on the nervous system.

In the comparison between ALS patients and HC, ALS patients demonstrate a highly significant elevation in CHI3L1 levels in CSF. In the control group with AMDS, the differential levels of CHI3L1 were superior to ONDS. Serum levels of CHI3L1 were lower in ALS patients compared to AMDS controls, but the difference was not statistically significant, possibly due to the limited number of studies included. CHI3L1 shows a strong upregulation during the late stages of macrophage differentiation [46]. Bonneh-Barkay et al. [47] demonstrated that CHI3L1 is associated with chronic neuroinflammation. Neuroinflammatory diseases exhibit significant in vivo expression of CHI3L1 through reactive astrocytes instead of macrophages/microglia. Additionally, the study revealed that pro-inflammatory mediators released by macrophages induce CHI3L1 transcription in astrocytes. Huang et al. have shown that in transgenic rat models, mutation of TDP-43 in astrocytes leads to the downregulation of neurotrophic genes and the upregulation of CHI3L1. Additionally, synthesized CHI3L1 selectively kills cortical neurons dose-dependently [48]. He et al. discovered that CHI3L1 can bind to interleukin 13 receptor α2 (IL-13Rα2) and plays a crucial role in CHI3L1 effector responses. Upon binding to IL-13Rα2, CHI3L1 activates MAPK/ERK, AKT/PKB, and Wnt/β-catenin signaling pathways, which in turn leads to the regulation of oxidative damage, apoptosis, pyroptosis, inflammasome activation, antibacterial response, and TGF-β1 production [49]. Connolly et al. proposed the hypothesis that CHI3L1, functioning as a signaling molecule, exerts multiple effects and mediates various neuroinflammatory responses and functional impairments in brain cells, thereby promoting neurodegeneration and triggering degenerative diseases of the nervous system, encompassing ALS and Alzheimer’s disease (AD). As research progresses, the association between ALS and CHI3L1 begins to unfold, highlighting the promising prospects of studying CHI3L1 as a biomarker for ALS [50].

CHI3L2 is a 39-kDa protein obtained from the conditioned medium of primary cultures of human articular chondrocyte [51]. Although there is limited research on CHI3L2, its expression has been identified in chondrocytes, synovial cells, and activated “M2” macrophages [52]. Elevated CHI3L2 expression has been noted in articular chondrocytes affected by osteoarthritis, indicating its potential utility as a biomarker for this condition [52]. The functionality of CHI3L2 is linked to immune response and tissue remodeling. The study by Sanfilippo et al. [53] revealed that, in contrast to the HC group, individuals diagnosed with spinal muscular atrophy displayed a notable increase in the expression levels of CHI3L1 and CHI3L2 within the motor cortex. Furthermore, their expression levels exhibited a negative correlation with survival duration. Mechanistic investigations regarding the interplay between CHI3L2 and ALS remain limited and require further exploration.

The constraints of this meta-analysis encompass the following aspects: Firstly, our meta-analysis faces a major constraint attributed to the inherent heterogeneity within the studies encompassed. Furthermore, potential heterogeneity sources may manifest in aspects like patient selection criteria and classification of control groups. It is worth noting that ALS is recognized as a heterogeneous condition encompassing motor and non-motor impairments. Secondly, despite our diligent efforts to acquire essential absent data from the authors, we received limited responses. Therefore, we used established methods to transform the data, as described earlier. Lastly, the included studies still need to be expanded, with fewer reports on blood-related aspects. The research on chitinases levels in the blood is relatively minor, and the same applies to CHI3L2. These factors make it challenging to conduct further analysis on these aspects.

We are the inaugural researchers to undertake a meta-analysis exploring chitinases as a potential diagnostic and prognostic biomarker for ALS. Based on our study, early supportive data suggest that chitinases may be a promising disease biomarker for ALS. Considering the existing absence of diagnostic and prognostic biomarkers for this condition, this outcome distinctly underscores the necessity for additional research into its applicability. In clinical practice, typical early diagnosis involves excluding other neurodegenerative diseases, especially diseases that mimic ALS. Our study results also happen to indicate that CSF CHIT1 and CHI3L1 have the potential to distinguish between ALS and AMD. As a result, there is a pressing demand for biomarkers that can aid and direct clinical decision-making, monitoring the disease progression, and evaluate the impacts of pharmaceutical interventions in clinical trials. These results open new perspectives for exploring chitinases as biomarkers and their functional relevance in ALS.

### Supplementary Information

Below is the link to the electronic supplementary material.

Supplementary file1 (EPS 21 KB)

Supplementary file2 (EPS 23 KB)

Supplementary file3 (EPS 360 KB)

Supplementary file4 (EPS 20 KB)

Supplementary file5 (EPS 17 KB)

Supplementary file6 (EPS 24 KB)

Supplementary file7 (EPS 20 KB)

Supplementary file8 (EPS 17 KB)

Supplementary file9 (EPS 17 KB)

Supplementary file10 (EPS 16 KB)

Supplementary file11 (EPS 20 KB)

Supplementary file12 (DOCX 45 KB)

Supplementary file13 (DOCX 32 KB)

## Data Availability

The data supporting the findings of this study can be obtained by making a reasonable request to the corresponding authors, Jing Zhou and Xinhong Zhu.
