# Supplementary material for: Chitinases as a potential diagnostic and prognostic biomarker for amyotrophic lateral sclerosis: a systematic review and meta-analysis
Source: Neurol Sci. 2024 Jan 9;45(6):2489–503. doi: 10.1007/s10072-024-07301-5 (PMC11081993; doi:10.1007/s10072-024-07301-5)
Supplement: Supplementary file 12 — Supplementary file12 (DOCX 45 KB) [file 10072_2024_7301_MOESM12_ESM.docx]

**Identification of studies via databases and registers**

Records identified n=233

PubMed (n =45)

web of science (n =37)

Cochrane library (n=7)

Embase (n=61)

Scopus (n=83)

Duplicate records removed (n =111)

**Identification**

Records excluded

Review articles (n=22)

Conference articles (n=28)

Editorial, letter and note (n=8)

Others(n=3)

Records screened by title and abstract (n = 122)

Records assessed for full-text screening (n =61)

No relevant articles (n=34)

Meta-analysis (n=3)

**Screening**

Reports excluded with reasons:

No relevant data (n =11)

Reports assessed for eligibility (n =24)

Studies included in qualitative and quantitative synthesis (n =13)

**Included**

*Consider, if feasible to do so, reporting the number of records identified from each database or register searched (rather than the total number across all databases/registers).

**If automation tools were used, indicate how many records were excluded by a human and how many were excluded by automation tools.

*From:*  Page MJ, McKenzie JE, Bossuyt PM, Boutron I, Hoffmann TC, Mulrow CD, et al. The PRISMA 2020 statement: an updated guideline for reporting systematic reviews. BMJ 2021;372:n71. doi: 10.1136/bmj.n71

For more information, visit: <http://www.prisma-statement.org/>
